# Effects of Fructose and Stress on Rat Renal Copper Metabolism and Antioxidant Enzymes Function

**DOI:** 10.3390/ijms23169023

**Published:** 2022-08-12

**Authors:** Danica Tasić, Miloš Opačić, Sanja Kovačević, Aleksandra Nikolić Kokić, Milena Dimitrijević, Dušan Nikolić, Danijela Vojnović Milutinović, Duško Blagojević, Ana Djordjevic, Jelena Brkljačić

**Affiliations:** 1 Department of Biochemistry, Institute for Biological Research “Siniša Stanković”, National Institute of Republic of Serbia, University of Belgrade, 142 Despot Stefan Blvd, 11060 Belgrade, Serbia; 2 Department of Life Sciences, Institute for Multidisciplinary Research, University of Belgrade, 142 Despot Stefan Blvd, 11060 Belgrade, Serbia; 3 Department of Physiology, Institute for Biological Research “Siniša Stanković”, National Institute of Republic of Serbia, University of Belgrade, 142 Despot Stefan Blvd, 11060 Belgrade, Serbia; 4 Department of Biology and Inland Waters Protection, Institute for Multidisciplinary Research, University of Belgrade, Kneza Višeslava 1, 11030 Belgrade, Serbia

**Keywords:** fructose-fed rat, kidney, copper transporter, copper chaperone of superoxide dismutase, oxidative stress

## Abstract

The effects of a fructose-rich diet and chronic stress on copper metabolism in the kidneys are still understudied. We investigated whether fructose and/or chronic unpredictable stress modulate copper metabolism in a way that affects redox homeostasis, thus contributing to progression of metabolic disturbances in the kidney. We determined protein level of copper transporters, chaperones, and cuproenzymes including cytochrome c oxidase, as well as antioxidant enzymes function in the kidneys of male Wistar rats subjected to 20% liquid fructose supplementation and/or chronic stress. Liquid fructose supplementation increased level of copper chaperone of superoxide dismutase and decreased metallothionein level, while rendering the level of copper importer and copper chaperones involved in copper delivery to mitochondria and trans Golgi network unaffected. Stress had no effect on renal copper metabolism. The activity and expression of renal antioxidant enzymes remained unaltered in all experimental groups. In conclusion, fructose, independently of stress, decreased renal copper level, and modulated renal copper metabolism as to preserve vital cellular function including mitochondrial energy production and antioxidative defense, at the expense of intracellular copper storage.

## 1. Introduction

Increased consumption of added sugar, especially fructose, as one of the major characteristics of modern lifestyle, along with chronic exposure to stress and lack of physical activity, poses a risk to human health [1,2]. Studies on animals and humans suggest that a high-fructose diet may be related to the pathophysiology of metabolic disorders such as obesity, insulin resistance-related disorders, nonalcoholic fatty liver disease, cardiovascular diseases, kidney injury, etc. [3,4,5,6].

Due to specificities in fructose metabolism, such as bypassing glycolytic regulatory enzyme phosphofructokinase, fructose overconsumption can cause alterations in lipid metabolism, glucose metabolism, and energy sensing [2,5,7]. Oxidative stress is considered as one of the underlying factors contributing to development of metabolic disturbances [8], and increased fructose consumption was found to affect cellular redox homeostasis and to induce low grade inflammation, thereby contributing to the pathophysiology [7,9,10]. In addition, fructose was found to affect absorption of redox-active transition metals including copper [11,12], and recent evidence suggests that inadequate copper intake may be related to pathogenesis of nonalcoholic fatty liver disease, obesity, metabolic syndrome, and diabetic cardiomyopathy [13,14,15,16]. However, the relation between fructose consumption, renal copper metabolism and redox homeostasis in the kidney is still understudied.

Copper is the third most abundant transition metal in humans, and copper-binding proteins comprise approximately 1% of the total eukaryotic proteome [17]. The propensity of copper to cycle between the two oxidation states, Cu(I) (cuprous ion) and Cu(II) (cupric ion), endows it with the ability to act as either a recipient or a donor of electrons, and as such, to frequently serves as a cofactor in many diverse physiological processes including antioxidant defense, mitochondrial energy production, neuropeptide processing machinery, pigmentation, extracellular matrix stability, etc. [18,19]. Many of the symptoms associated with copper deficiency can be attributed to a decreased activity of copper-dependent enzymes [20]. At the same time, the capacity to shift between oxidation states can also enable free copper ions to participate in the generation of reactive oxygen species via a Fenton-like reaction, thereby inducing damage of cellular macromolecules including proteins, lipids, and nucleic acids [21,22]. Under conditions of copper overload, free copper ions may exert toxic effects by inducing oxidative stress (specifically by catalyzing the formation of reactive oxygen species and decreasing glutathione levels) [22]; and also by displacing other metals from their cognate ligands in metalloproteins, which results in improper protein conformation and impaired function [23]. Consequently, copper metabolism is tightly regulated both at a systemic and cellular level [24,25].

Mammals acquire copper from dietary sources, and the absorption mainly takes place in the duodenum [19]. Enterocytes import copper at the apical membrane mainly using copper transporter protein-1 (CTR1), and release it from the basolateral membrane into the bloodstream using copper-transporting P-type ATPase 7A (ATP7A). The majority of absorbed copper initially reaches the liver via the portal system. The liver is the main regulatory organ of copper homeostasis as it governs the distribution of copper to the peripheral tissues (including kidney); and regulates the removal of copper excess by biliary excretion [26], since urinary excretion plays a minor role (<0.1 mg/day) [19,27]. Secreted from hepatocytes, copper is predominantly bound to ceruloplasmin, and as such it reaches peripheral tissues via bloodstream to be utilized as a cofactor for universally expressed cuproenzymes such as cytochrome c oxidase (COX) and superoxide dismutase (SOD), and tissue-specific cuproenzymes such as lysyl oxidase in connective tissue, dopamine monooxygenase in brain and tyrosinase in melanocytes [19,25]. Nevertheless, the regulation of intracellular copper distribution is thought to be similar in all tissues. Namely, copper ions are imported into peripheral tissue cells by CTR1, and afterwards either sequestrated and stored in a complex with metallothioneins (MT), or distributed to target copper-binding proteins using a system of copper chaperones such as: copper chaperone of superoxide dismutase (CCS), cytochrome c oxidase copper chaperone 17 (COX17), cytochrome c oxidase assembly factors (SCO) and antioxidant 1 copper chaperone (ATOX1) [28]. Copper is structural and catalytic cofactor of COX, whose subunits I and II (COX1 and COX2) contain copper centers CuB and CuA. COX17 escorts copper from the cytoplasm to the mitochondrial lumen and delivers it to COX-assembly chaperones that facilitate copper insertion into COX [29]. CCS delivers copper to the antioxidant enzyme Cu/Zn SOD (SOD1) [30], and ATOX1 escorts copper to the trans-Golgi network and delivers it to ATP7A, thus enabling incorporation of copper into newly synthesized cuproproteins, and, in enterocytes and hepatocytes to ATP7A and ATP7B, thereby enabling copper excretion into the blood or bile [31].

Increased fructose consumption and chronic exposure to stress usually go hand-in-hand in everyday life, and both contribute to the development and progression of metabolic disorders [1]. Oxidative stress is one of the underlying molecular mechanisms contributing to progression of metabolic disturbances [8], and both chronic stress and fructose can alter the redox status of the cell [32,33,34,35,36,37,38,39,40,41]. The majority of previous studies investigating effects of dietary modulations on copper metabolism were focused on the liver as the main regulatory organ of copper homeostasis, while less is known about the effects of fructose, stress and their combination on renal copper metabolism. Also, the relation between renal copper metabolism and redox homeostasis in the kidney of fructose-fed stressed rats has not been investigated yet.

Our previous observation that fructose enhances renal fructolysis and affects glucose and lipid metabolism in the rat kidney [42] prompted us to investigate whether liquid fructose supplementation affects renal copper metabolism and antioxidant enzymes function in a way that might contribute to progression of metabolic disturbances in the kidneys and to learn whether chronic stress potentiates aggravating effects of fructose. To that end, we measured the protein level of copper transporters and chaperones, copper-binding proteins and cuproenzymes, as well as protein level and activity of antioxidant enzymes: SOD1, mitochondrial MnSOD (SOD2), catalase (CAT), glutathione peroxidase (GPX) and glutathione reductase (GR), in the kidneys of male rats subjected to liquid fructose supplementation and/or chronic unpredictable stress. The results of this study show that liquid fructose supplementation irrespectively of stress decreases renal copper level, while plasma copper level remains unaltered. In addition, fructose diet modulates copper metabolism in the kidney by reducing MT level, i.e., cellular copper reserves, and increases CCS level which goes in line with fructose-induced copper deficiency in the kidney. However, the sustainability of vital cellular processes including mitochondrial energy production and antioxidative defense was preserved.

## 2. Results

### 2.1. Effects of Fructose and Stress on Plasma Glucose, Insulin and Copper Levels

As shown in Table 1, liquid fructose supplementation significantly increased plasma glucose (F_1,20_ = 4.4, *p* < 0.01) and HOMA index (F_1,20_ = 6.9, *p* < 0.05). None of the treatments affected plasma copper and insulin levels (Table 1).

### 2.2. Effects of Fructose and Stress on Renal Copper Metabolism

**Figure 1:**
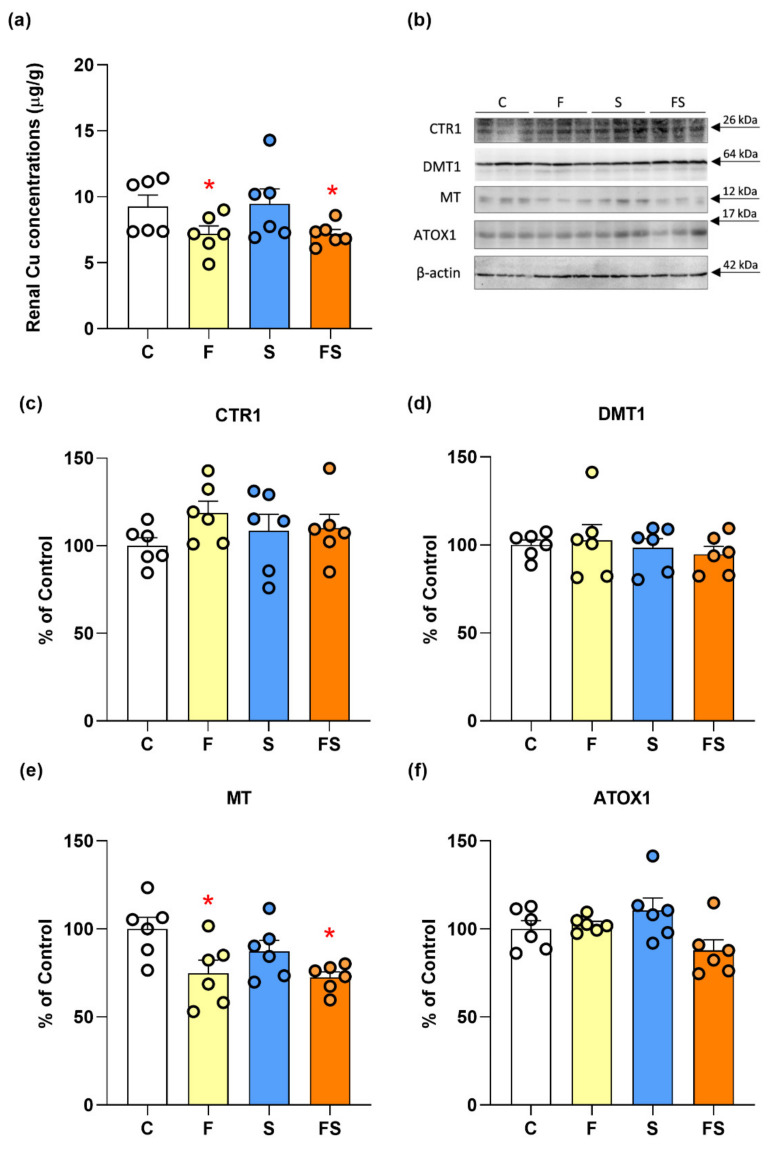
Effects of liquid fructose supplementation and/or stress on copper concentration and proteins involved in copper import (CTR1 and DMT1), storage (MT), and transport to Golgi network (ATOX1). Groups: control (C), fructose-fed (F), stress (S) and fructose + stress (FS). Renal copper concentrations (**a**), representative Western blots (**b**) and protein level of CTR1 (**c**), DMT1 (**d**) MT (**e**) and ATOX1 (**f**) in renal whole cell extracts. Scatter plot with bar graphs represent the means ± SEMs for each protein normalized to *β*-actin and expressed relative to controls (*n* = 6 animals/group). Two-way ANOVA was used to evaluate the effects of fructose and stress, and their interaction. Asterisk denotes statistically significant main effect of fructose. *p* < 0.05.

Liquid fructose supplementation decreased the level of copper in the kidney (F_1,20_ = 27.1, *p* < 0.05) (Figure 1a). There was no S × F interaction. Liquid fructose supplementation and stress, applied alone or in combination, had no statistically significant effect on protein level of copper importer CTR1 (Figure 1c). Also, the level of divalent metal transporter (DMT1) remained unaltered (Figure 1d). Once inside the cell, copper is escorted to specific compartments by chaperones, or stored in a complex with MT. Liquid fructose supplementation decreased MT level (F_1,20_ = 10.9, *p* < 0.01) (Figure 1e). There was no S × F interaction. At the same time, the level of ATOX1, which delivers copper for metalation and secretory pathway in the trans-Golgi network, remained unaltered (Figure 1f).

**Figure 2:**
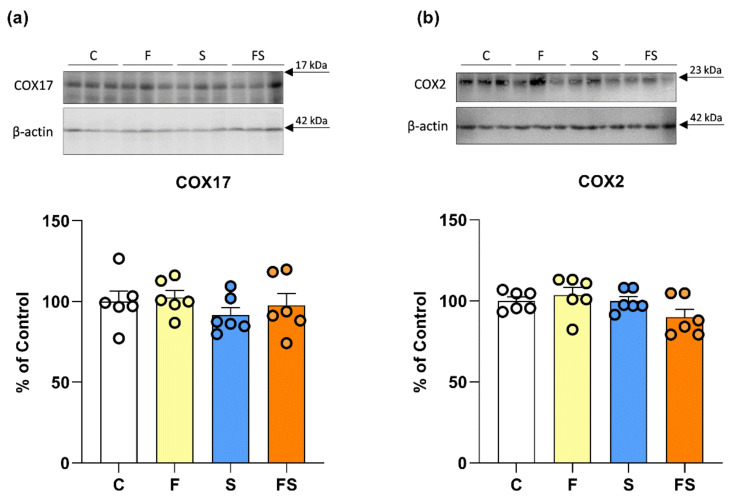
Effects of liquid fructose supplementation and/or stress on COX17 and COX2 protein level. Groups: control (C), fructose-fed (F), stress (S) and fructose + stress (FS). Protein level of COX17 (**a**) and COX2 (**b**) in renal whole cell extracts was measured by Western blot. Scatter plot with bar graphs represent the means ± SEMs for each protein normalized to *β*-actin and expressed relative to controls (*n* = 6 animals/group). Two-way ANOVA was used to evaluate the effects of fructose and stress, and their interaction.

None of the treatments affected the level of COX17, involved in the recruitment and delivery of copper to mitochondria (Figure 2a), and the level of cuproenzyme COX2 (Figure 2b).

**Figure 3:**
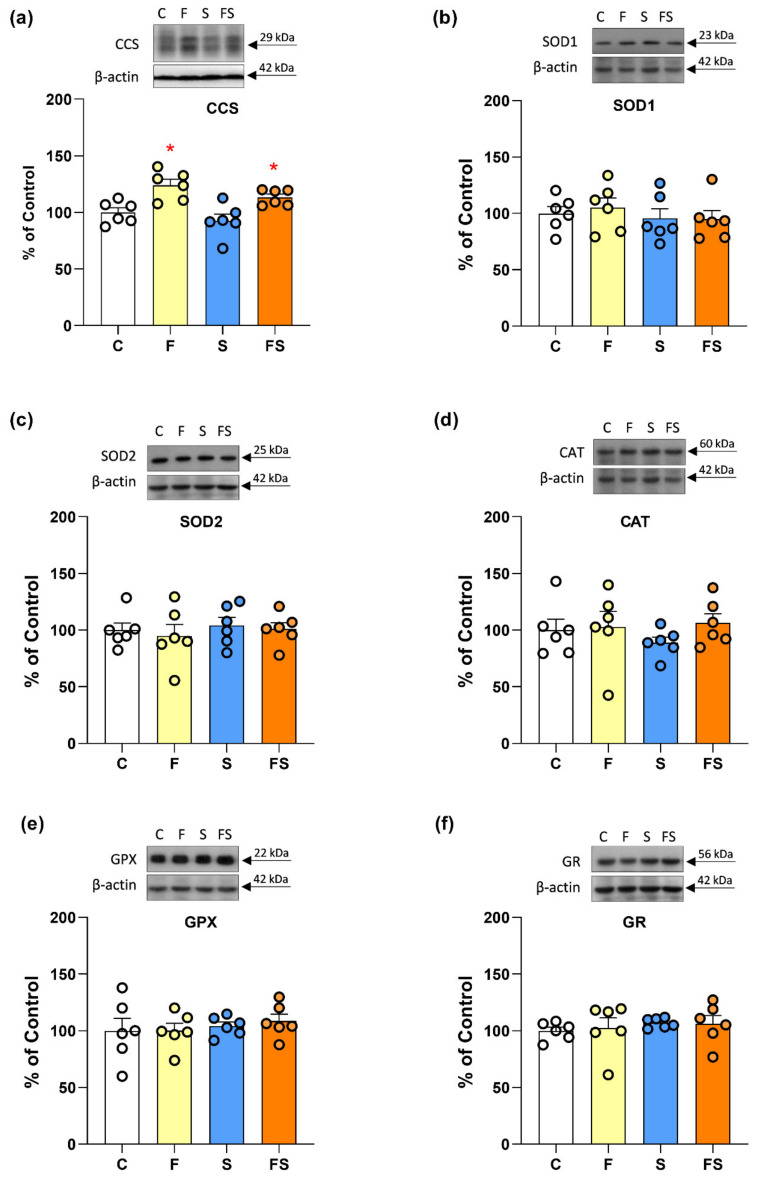
Effects of liquid fructose supplementation and/or stress on the level of copper chaperone CCS and antioxidant enzymes. Groups: control (C), fructose-fed (F), stress (S) and fructose + stress (FS). Protein level of CCS (**a**), SOD1 (**b**) and SOD2 (**c**), CAT (**d**), GPX (**e**) and GR (**f**) in renal whole cell extracts, was measured by Western blot. Scatter plot with bar graphs represent the means ± SEMs for each protein normalized to *β*-actin and expressed relative to controls (*n* = 6 animals/group). Two-way ANOVA was used to evaluate the effects of fructose and stress, and their interaction. Asterisk denotes statistically significant main effect of fructose. *p* < 0.05.

Liquid fructose supplementation increased the level of CCS (F_1,20_ = 26.15, *p* < 0.001) (Figure 3a) while the level of SOD1 remained unaltered (Figure 3b). No S × F interaction was observed.

### 2.3. Effects of Fructose and Stress on Renal Antioxidant Enzymes Function

Neither dietary fructose nor stress appeared to affect antioxidant enzyme function. Namely, there were no significant effects of fructose or stress on the protein level of SOD2, CAT, GPX and GR (Figure 3c–f). Also, the activities of antioxidant enzymes were not affected by any of the treatments (Table 2).

## 3. Discussion

Chronic stress and increased fructose consumption were shown to affect glucose and lipid metabolism in rats in a tissue-specific manner [39,42,43,44]. Using the same animal model herein, we have previously observed that liquid fructose supplementation up-regulates fructose uptake and fructolysis in the kidney [42]. Fructose carbons in the kidney were subsequently utilized for gluconeogenesis and de novo lipogenesis [42], and all effects of fructose were independent of stress. In the current study, we report that copper metabolism in the kidney is exclusively modulated by fructose supplementation, without significant contribution of stress. At the same time, the sustainability of vital cellular processes including mitochondrial energy production and antioxidative defense was preserved in all experimental groups.

A high-fructose diet was shown to induce a majority of signs of the metabolic syndrome, including increased blood glucose, glucose intolerance, insulin resistance, hypertriglyceridemia, and dyslipidemia [45], all of which, in the long term, contribute to development of metabolic disorders including type 2 diabetes and cardiovascular diseases [4]. In line with this, our current results show that liquid fructose supplementation increases blood glucose level and disturbs insulin sensitivity, as judged by the increased HOMA index. One of the most consistently reported effects of increased fructose consumption is hypertriglyceridemia, and using the same animal model herein, we have previously reported that liquid fructose supplementation increases plasma triglycerides.

Fructose was found to affect copper absorption [11], and recent evidence points to copper–fructose interactions as important contributors in development and progression of metabolic syndrome and nonalcoholic fatty liver disease [46]. Although underlying molecular mechanisms are not fully understood, previous studies imply the upregulation of hepatic fatty acid synthase (FAS) and sterol regulatory element-binding protein-1 (SREBP-1) [14,47,48]. Fructose can also upregulate renal FAS expression and induce lipid accumulation in the kidney [49], however the possible role of copper–fructose interactions in the kidney are not fully investigated. We have previously reported that liquid fructose supplementation led to an increase in FAS expression and affected fatty acid profiles in the kidney, although these changes were not accompanied by intrarenal lipid accumulation [42]. Current results showing decreased copper level in the kidney of both fructose-fed groups might imply a possible relation of copper status with fructose-induced alterations of renal lipid metabolism. However, further investigation along these lines is necessary.

Fructose overconsumption was linked to copper deficiency in both animals and humans [14]. Besides decreasing the intake of solid food [42,50], fructose was shown to directly affect copper bioavailability and inhibit duodenal copper absorption by targeting intestinal CTR1 [14,51]. Song et al. [14] have reported that 30% liquid fructose supplementation decreases hepatic copper level, while rendering plasma copper level unaltered. In line with these, our results show that 20% fructose supplementation also has no effect on plasma copper level. At the same time, renal copper level was decreased in both fructose-fed groups, and this effect was independent of stress. Copper content in the kidney is among the highest in the body [19], and appears to be precisely regulated. Bioavailability of intracellular copper is tightly controlled by copper transporters, chaperones, and storage proteins [24]. Copper ions are imported into kidney by CTR1 and by DMT1. Since both proximal and distal tubular cells express CTR1 [52], glomerular-filtered copper can be reabsorbed from the urine when blood copper levels are low [53], implying that increased renal CTR1 level in mice on a low copper diet might compensate for the decrease in copper supply [53]. Furthermore, CTR1 is an integral membrane protein, which can be rapidly internalized in the presence of elevated copper, and recycled back to the plasma membrane when extracellular copper is removed [54]. In the current study, neither stress nor fructose had a statistically significant effect on CTR1 protein level in renal whole cell extracts. DMT1, the predominant iron importer, also transports other metal ions, including copper. In this study, the level of DMT1 also remained unaltered.

Once inside the cell, copper is either stored in complex with MT, or distributed via cytosolic, mitochondrial, and Golgi routes to cuproenzymes. In the cytosol, CCS delivers copper to SOD1. CCS concentration is modulated in response to changes in cellular copper status at the post-translational level through degradation by the 26S proteasome complex [55]. When cellular copper concentration is low, CCS level is high, and vice versa, in the presence of elevated copper, CCS level is low [55,56,57]. As CCS up-regulation represents one of the most robust changes specific for copper deficiency, CCS level could reflect cellular copper status [56,57,58]. Indeed, CCS was suggested as the most promising new potential biomarker for copper deficiency as well as copper excess [59,60]. In support of this, our results showing increased CCS level in both fructose-fed groups go in line with the observed decrease in renal copper level. At the same time SOD1 protein level and enzyme activity remained unaltered. Stress had no modulatory effect on renal copper status. Although CCS supplies SOD1 with copper, the levels of SOD1 and CCS do not necessarily match, which could be attributed to multiple different levels of SOD1 regulation [61,62]. Increased CCS level and unaltered SOD1 expression and activity, observed herein, suggest that despite decreased copper levels in the kidney, a sufficient amount of copper is allocated to SOD1. Moreover, the activity and expression of other antioxidant enzymes SOD2, CAT, GPX and GR also remained unaltered in all experimental groups, suggesting that fructose and stress had no effect on antioxidant defense system in the kidney. In contrast to our results, previous studies have shown that 60% fructose-rich diet induces oxidative stress in the rat kidney [63,64]. The discrepancies between the studies could be attributed to the dose (20% vs. 60% fructose) and duration of the treatment (8 vs. 10–12 weeks).

The mitochondrial respiratory chain is the primary source of cellular reactive oxygen species [65]. Besides high activity of the electron transfer chain and highly polarized transmembrane potential in highly oxidative tissues such as the kidney, an electron transport dysfunction or imbalance in complexes also contribute to the production of reactive oxygen species. A low copper diet was shown to downregulate the COX level in rat hearts [58,66]. However, in our study, the expression and activity of mitochondrial antioxidant enzyme SOD2, as well as SOD1, which partially localizes to the mitochondrial intermembrane space, remained unchanged in all treated groups, which goes in line with unaltered levels of the copper-containing COX-subunit 2. An unaltered level of COX17 implies continuous copper supply to the mitochondrial matrix, which serves as a pool for COX metalation. Although fructose decreased renal copper status, vital cellular processes such as antioxidative defense and mitochondrial energy production appear to be preserved.

ATOX1 delivers copper to ATP7A located in the trans-Golgi network, thus enabling maturation of newly synthesized cuproproteins within the secretory pathway [20]. Once assembled, cuproproteins are secreted out of the cell or are delivered to specific organelles. Kidneys synthesize several cuproenzymes including extracellular Cu/Zn SOD (SOD3), diamine oxidase, and ferroxidase [67,68,69]. Judging by unaltered ATOX1 level, we could assume that a sufficient amount of copper is delivered to the Golgi network for metalation. Although kidneys express ATP7B, copper urinary excretion is low. When cellular copper level is high, ATP7B relocates to the plasma membrane and participates in copper export. Since in our experimental setting liquid fructose supplementation reduced renal copper content, we could assume that the majority of copper in the trans-Golgi network is incorporated into newly synthesized cuproproteins, rather than excreted. Nevertheless, as the intracellular localization and function of ATP7A and ATP7B depend on the amount of copper, further immunohistochemical analysis could provide deeper insight into the possible modulation of their function by fructose. Finally, since ATOX1 plays an important role in antioxidant defense and its expression is upregulated by reactive oxygen species [70], its unaltered level supports the observation of oxidative stress absence in the kidney of both fructose-fed groups.

The excess of intracellular copper is sequestered by MTs, which act as buffer to prevent the production of reactive oxygen species [71]. MTs are a family of low molecular weight proteins that contain 20 cysteine residues in their amino acid sequence, which enable them to chelate a large portion of metal ions and participate in zinc and copper homeostasis as well as in metal detoxification. MT-1 and MT-2 are ubiquitously expressed in several organs including the liver, kidney and intestine, and their expression can be upregulated by metal ions, including copper, zinc and toxic heavy metals such as cadmium [72,73]. At the same time, metal deficiency can downregulate tissue MT level [74]. Rats fed with a zinc-deficient diet had significantly decreased MT level in the liver [75]. Also, a decrease in renal MT protein was reported in rats subjected to a copper-deficient diet [76]. A decreased level of MT observed in both fructose-fed groups suggests that renal cells reduce intracellularly stored copper. Since soluble copper chaperones (CCS, COX17, and ATOX) compete for intracellular copper in order to facilitate supply to their specific target compartments, it is possible to assume that liquid fructose supplementation modulates renal copper metabolism in a way that enables sustainability of vital function dependent on adequate copper supply to cuproenzymes such as COX and SOD1, at the expense of copper storage capacities.

As mentioned above, stress had no effect on renal copper status, despite its potential influence on various micronutrients in the body and capability to induce oxidative stress in various tissues [33,34,35,36,37,38]. Indeed, the current results show the absence of stress-related alterations in antioxidant enzymes function, which could be attributed to higher antioxidant capacity of the kidney [77]. Overall, the results of this study show that liquid fructose supplementation modulates copper metabolism in the kidney, while in the combined treatment no interaction between fructose and stress was found, and stress alone had no effect on renal copper status.

In conclusion, liquid fructose supplementation, independently of stress, decreased renal copper level, as evidenced by increased CCS, and reduced MT level. The results show the absence of oxidative stress, unaltered SOD1 function and COX2 protein level, suggesting that modulation of renal copper metabolism is directed to provide enough copper to sustain vital cellular function at the expense of its storage. However, we could assume that prolonged fructose supplementation could ultimately increase copper turnover, deplete cellular copper reserves and lead to dysfunction of the electron transport chain and antioxidant defense, and induce tissue damage.

## 4. Materials and Methods

### 4.1. Animals and Treatment

Male Wistar rats, bred in our laboratory, were randomly divided into four experimental groups (*n* = 6 rats/group): control (C), fructose (F), stress (S) and fructose + stress (FS) group. Animals were kept under standard conditions, at 22 °C with a 12-h light/dark cycle. As described previously [78], all rats were fed ad libitum with commercial rat food (Laboratory Rat Food R20: 20% protein, 62.6% carbohydrate and 3.2% fat, mineral and vitamin mix; Veterinary Institute Subotica, Serbia). F and FS groups had 20% (*w*/*v*) fructose solution instead of drinking water during 9 weeks. S and FS groups were subjected to chronic unpredictable stress protocol (modified from [79]) during the last 4 weeks of the treatment. The stress protocol included following daily stressors: forced swimming in cold water for 10 min, physical restraint for 60 min, exposure to a cold room (4 °C) for 50 min, wet bedding for 4 h, switching cages for 2 h, rocking cages for 1 h, and cage tilt (45°) overnight. The unpredictability of the stressors (type of daily stressor(s), number of stressor(s) (1 or 2) and the onset of stress exposure (between 9 AM and 4 PM) was achieved through random selection at the beginning of the treatment. A particular stressor was never applied on 2 consecutive days or twice per day.

All animal procedures were in compliance with Directive 2010/63/EU on the protection of animals used for experimental and other scientific purposes, and were approved by the Ethical Committee for the Use of Laboratory Animals of the Institute for Biological Research “Siniša Stanković”, University of Belgrade (No. 02-11/14).

### 4.2. Biochemical Analysis

After the end of the treatment, animals were killed by rapid decapitation (Harvard Apparatus, Holliston, MA, USA). EDTA containing tubes were used for trunk blood collection. Low speed centrifugation (1600× *g*/10 min) was used for plasma preparation, which was stored at −20 °C for further processing.

The concentrations of glucose (Cat. number GL8038, Randox Laboratories Ltd., Crumlin, UK) and insulin (Cat. number 90060, Crystal Chem, Elk Grove Village, USA) were measured in the plasma samples by commercial kits using the semi-automatic biochemistry analyzer Rayto 1904-C (Rayto, Guangdong Province, Shenzhen, Nanshan, China). Insulin sensitivity was evaluated by homeostasis model assessment (HOMA) index calculation using the formula: insulin (mU/L) × (glucose (mmol/L)/22.5).

### 4.3. Tissue Preparation

The kidneys were quickly excised, weighed, and stored in liquid nitrogen until use. After thawing, the tissue was homogenized in ice cold RIPA buffer (50 M Tris-HCl pH 7.2, 1 mM EDTA, 150 mM NaCl, 0.1% sodium dodecyl sulphate (SDS), 1% Nonidet P-40, 0.5% sodium deoxycholate, 2 mM DTT, protease, and phosphatase inhibitors). The homogenates were sonicated (3 × 5 s, 1 A, 50/60 Hz), incubated for 60 min on ice with continuous agitation and frequent vortexing, and centrifuged (16,000× *g*, 20 min, 4 °C). All steps were conducted at 0–4 °C and all samples were stored in liquid nitrogen.

### 4.4. SDS-Polyacrylamide Gel Electrophoresis (SDS-PAGE) and Immunoblotting

After boiling in Laemmli′s sample buffer, proteins (40 μg) were resolved on 7%, 12% or 15% SDS-polyacrylamide gels, and transferred to the PVDF membrane. Membranes were blocked with 5% BSA, and incubated with primary antibody. CTR1, DMT1, ATOX1, COX17, COX2 and CCS were detected using Santa Cruz Biotechnology (Santa Cruz, CA, USA) antibodies: sc-18473, sc-166884, sc-398742, sc-393617, sc-514489, sc-55561, respectively. SOD1, SOD2, CAT, GR and GPX, were detected using Abcam (Trumpington, Cambridge, UK) antibodies: ab13498, ab13533, ab16731, ab16801, ab22604, respectively. Anti-MT-1/MT-2 antibody (M0639) was the product of Dako (Agilent, Santa Clara, CA, USA). β-actin was detected by AC-15 antibody (Sigma-Aldrich, St. Louis, MO, USA). The blots were incubated with anti-rabbit, anti-goat, or anti-mouse secondary antibodies conjugated with horseradish peroxidase. Immunoreactive proteins were visualized by the enhanced chemifluorescence method. Quantitative analysis of immunoreactive bands was done using iBright^TM^ FL1500 Imaging System Software (Thermo Fisher Scientific, Waltham, MA, USA). All experimental samples and controls used for one comparative analysis are run on the same blot/gel.

### 4.5. Antioxidant Enzymes Activity

For determination of antioxidant enzyme activity, tissue was homogenized in 10 vol. (*w*/*v*) of buffer (50 mM Tris, 0.25 M sucrose, 0.1 mM EDTA, pH 7.4) and sonicated (3 × 10 s at 10 MHz on ice) prior to 60 min centrifugation at 105,000× *g*. The supernatants were used to measure SOD1, SOD2, CAT, GPX, and GR activities spectrophotometrically, as described previously [80]. In brief, total SOD activity was determined by the adrenaline method [81]. One SOD unit was defined as the amount of the enzyme necessary to decrease the rate of adrenalin auto-oxidation by 50% at pH 10.2. For measurement of SOD2 activity, the assay was performed after preincubation with 8 mM potassium cyanide. SOD1 activity was calculated as the difference between the total SOD and SOD2 activities. CAT activity was determined using the method of Beutler [82]. One unit of CAT activity was defined as the amount of the enzyme that decomposes 1 mmol H_2_O_2_ per minute at 25 °C and pH 7.0. GPX activity was determined by the glutathione reduction of t-butyl hydroperoxide, using a modification of the assay described by Paglia and Valenine [83]. One GPX unit was defined as the amount of the enzyme needed to oxidize 1 mmol NADPH per minute at 25 °C and pH 7.0. The activity of GR was determined by the method of Glatzle et al. [84], and one unit of GR activity was defined as the amount of the enzyme needed to oxidize 1 nmol NADPH per minute at 25 °C and pH 7.4. All enzyme activities are expressed as units (U) per mg of protein.

### 4.6. Determination of Copper Concentrations in Plasma and Kidney

Samples portions (0.4 mL of plasma and 0.5 g of kidney) were digested at the Blood program (180 °C) for blood plasma and Fresh kidney program (200 °C) for kidney, in a microwave digester (ETHOS EASY, Milestone, Italy), by adding 4 mL of hydrogen peroxide (30%) (Merck, Darmstadt, Germany) and 6 mL of nitric acid (65%) (Merck, Darmstadt, Germany). Analytical blank samples (four in total) were prepared to resolve the potential presence of analyzed elements in utilized reagents. Digested samples were diluted with distilled water to a total volume of 15 mL after cooling at room temperature.

Analysis of copper concentrations was performed by inductively coupled plasma spectrometry (ICP-OES, Avio 200, Perkin Elmer, Waltham, MA, USA) on the wavelength line for Cu 324.752.

### 4.7. Data Presentation and Analysis

Data are presented as means ± SEM. Effects of fructose and stress, and their interaction were analyzed by two-way ANOVA. Statistical analysis was performed using STATISTICA 8.0 software (StatSoft, Inc., Tulsa, OK, USA). A probability level of *p* < 0.05 was considered statistically significant.

## Figures and Tables

**Table 1:** Effects of liquid fructose supplementation and/or stress on plasma parameters.

Parameter	Control	Fructose	Stress	Fructose + Stress
Glucose (mmol/L)	5.68 ± 0.16	7.14 ± 0.33 **	6.34 ± 0.14	6.59 ± 0.29 **
Insulin (ng/mL)	2.47 ± 0.31	4.03 ± 0.50	3.33 ± 0.47	3.17 ± 0.55
HOMA	0.63 ± 0.09	1.28 ± 0.17 *	0.94 ± 0.14	1.03 ± 0.13 *
Cu (μg/g)	1.47 ± 0.08	1.48 ± 0.14	1.28 ± 0.06	1.33 ± 0.07

All data are presented as mean ± SEM (*n* = 6 animals per group). Two-way ANOVA was used to evaluate the effects of fructose and stress, and their interaction. Asterisk denotes statistically significant main effect of fructose. * *p* < 0.05; ** *p* < 0.01.

**Table 2:** Effects of liquid fructose supplementation and/or stress on antioxidant enzyme activity.

Enzyme	Control	Fructose	Stress	Fructose + Stress
SOD1	22.6 ± 1.1	24.3 ± 2.2	20.9 ± 1.8	21.8 ± 2.1
SOD2	1.3 ± 0.2	1.1 ± 0.1	1.3 ± 0.1	1.1 ± 0.2
CAT	63.7 ± 5.8	71.3 ± 2.4	75.7 ± 2.8	72.5 ± 2.4
GPX	377.7 ± 17.9	427.7 ± 29.1	397.1 ± 19.6	428.5 ± 15.2
GR	183.9 ± 14.5	195.3 ± 9.2	180.4 ± 19.6	201.4 ± 12.8

Antioxidant enzyme activities in renal whole cell extracts are expressed as units per milligram of protein. Values are expressed as the means ± SEM (*n =* 6 animals/group).

## Data Availability

The data presented in this study are available on request from the corresponding author.
